# Brachial-Ankle Pulse Wave Velocity is Associated with the Risk of New Carotid Plaque Formation: Data from a Chinese Community-based Cohort

**DOI:** 10.1038/s41598-018-25579-2

**Published:** 2018-05-04

**Authors:** Yao Yang, Fangfang Fan, Minghao Kou, Ying Yang, Guanliang Cheng, Jia Jia, Lan Gao, Zechen Zhou, Dafang Chen, Yan Zhang, Yong Huo

**Affiliations:** 1 Department of Cardiology, Peking University First Hospital, Beijing, China; 2 School of Public Health, Peking University Health Science Centre, Beijing, China

## Abstract

Artery stiffness is an independent marker for atherosclerotic cardiovascular diseases. However, whether the brachial-ankle pulse wave velocity (ba-PWV) is related to new carotid plaque formation is unresolved. This study aimed to investigate the association between baseline ba-PWV and new carotid plaque formation in a Chinese community-based population without carotid plaques at baseline. This study population consisted of a total of 738 participants from an atherosclerosis cohort in Beijing, China. After a mean 2.3-year follow-up, the incidence of carotid plaques were 21.2% and 36.5% in the groups with ba-PWV < 1,400 cm/s and ≥1,400 cm/s, respectively. Compared with baseline ba-PWV < 1,400 cm/s group, ba-PWV ≥ 1,400 cm/s group was significantly associated with the incidence of new carotid plaque formation (odds ratio [OR] = 2.14, 95% CI: 1.50–3.03, P < 0.01), even after adjusting for common risk factors (OR = 1.52, 95% CI: 1.02–2.25, P = 0.04). Furthermore, there was a strong relationship between baseline ba-PWV and carotid plaque formation in subjects with ba-PWV < 1,400 cm/s, but no such relationship was found in subjects with baseline ba-PWV ≥ 1,400 cm/s. In conclusion, this study suggests that baseline ba-PWV is independently associated with the risk of carotid plaque formation in a Chinese community-based population.

## Introduction

Arterial stiffness and reduction of arterial elasticity have been shown to be involved in the progression of atherosclerotic diseases^1^ through the stretch, phasic mechanical stress and shear stress imposed on vessels as well as endothelial dysfunction^2,3^.

Pulse wave velocity (PWV) is the most common method used to evaluate arterial stiffness and an independent marker of the presence of atherosclerotic cardiovascular diseases^4–6^. Brachial-ankle pulse wave velocity (ba-PWV), an index combining elastic and muscular peripheral arterial stiffness, is widely useful in cardiovascular outcome predictions^7–10^.

Carotid plaques are important indicators of carotid atherosclerosis and have been identified as important risk factors for ischaemic stroke^11^. New carotid plaque formation may be a marker of future carotid atherosclerotic disease. Previous studies have shown that an elevated ba-PWV is associated with cardiovascular disease (CVD), coronary artery calcification, intracranial arterial stenosis, stroke and the prognosis of stroke^12–16^. Since only a few cross-sectional studies have investigated the relationship between ba-PWV and the incidence of carotid atherosclerotic plaques in community-based subjects, cohort studies of this type are still warranted.

In this study, we hypothesized that ba-PWV was related to new carotid plaque formation, and we aimed to longitudinally assess the relationship between these two factors in a Chinese community-based population with no carotid plaques at baseline.

## Results

### Baseline characteristics of the study participants

Table 1 reports the demographic and clinical characteristics of the study population. We divided our subjects into two groups using the baseline ba-PWV value (ba-PWV < 1,400 cm/s, n = 259 and ba-PWV ≥ 1,400 cm/s, n = 479). The subjects (n = 738) were 49.8 ± 4.5 years old in the group with a PWV < 1,400 cm/s and 52.5 ± 4.8 years old in the group with a PWV ≥ 1,400 cm/s (P < 0.01). The percentages of males in the two groups were 18.1% and 33.6% (P < 0.01), respectively. The latter group was associated with a higher level of body mass index (BMI), systolic blood pressure, diastolic blood pressure, heart rate, fasting blood glucose (FBG), low-density lipoprotein cholesterol (LDL-C), triglycerides (TG), serum creatinine (SCR), a lower level of high-density lipoprotein cholesterol (HDL-C), and a higher ratio of, hypertension, diabetes mellitus, dyslipidemia, anti-hypertensive medication, lipid-lowering medication and anti-diabetic medication. No significant difference was found in the prevalence of tobacco intake, alcohol intake and the self-reported CVD history between the groups with a PWV < 1,400 cm/s and the group with a PWV ≥ 1,400 cm/s. (Table 1).

**Table 1:** Demographic and clinical characteristics at baseline (n = 738).

	Total	PWV < 1,400 cm/s (n = 259)	PWV ≥ 1,400 cm/s (n = 479)	P value
Age	51.6 ± 4.9	49.8 ± 4.5	52.5 ± 4.8	<0.01
Male, n (%)	208 (28.2%)	47 (18.1%)	161 (33.6%)	<0.01
Tobacco intake, n (%)	127 (17.2%)	37 (14.3%)	90 (18.8%)	0.12
Alcohol intake, n (%)	170 (23.0%)	54 (20.8%)	116 (24.2%)	0.30
BMI, kg/m^2^	25.7 ± 3.3	25.2 ± 3.1	25.9 ± 3.2	<0.01
Total cholesterol, mmol/L	5.29 ± 0.93	5.20 ± 0.91	5.33 ± 0.94	0.06
HDL-C, mmol/L	1.48 ± 0.39	1.56 ± 0.40	1.44 ± 0.39	<0.01
LDL-C, mmol/L	3.19 ± 0.77	3.10 ± 0.73	3.24 ± 0.79	0.01
Triglycerides, mmol/L*	1.58 ± 1.49	1.32 ± 1.05	1.72 ± 1.67	<0.01
FBG, mmol/L	5.89 ± 1.36	5.59 ± 1.06	6.06 ± 1.47	<0.01
Serum creatinine, μmol/L	56.5 ± 12.0	54.3 ± 9.3	57.7 ± 13.0	<0.01
Hypertension, n (%)	256 (34.7%)	27 (10.4%)	229 (47.8%)	<0.01
Systolic blood pressure, mmHg	128.9 ± 14.9	120.2 ± 11.5	133.6 ± 14.4	<0.01
Diastolic blood pressure, mmHg	75.4 ± 9.5	71.8 ± 7.8	77.4 ± 9.7	<0.01
Diabetes mellitus, n (%)	114 (15.4%)	22 (8.5%)	92 (19.2%)	<0.01
Heart rate, bpm	74.0 ± 11.4	71.2 ± 11.2	75.4 ± 11.2	<0.01
Dyslipidemia, n (%)	496 (67.2%)	154 (59.5%)	342 (71.4%)	<0.01
Anti-hypertension medicine, n (%)	146 (19.9%)	15 (5.8%)	131 (27.5%)	<0.01
Anti-diabetes medicine, n (%)	49 (6.6%)	9 (3.5%)	40 (8.4%)	0.01
Lipid-lowering medicine, n (%)	41 (5.6%)	6 (2.3%)	35 (7.4%)	<0.01
Self-reported CVD history, n (%)	49 (6.6%)	15 (5.8%)	34 (7.1%)	0.496
ba-PWV, cm/s	1,516 ± 263	1,263 ± 96	1,653 ± 219	<0.01

Notes: Abbreviations: BMI indicates body mass index; HDL-C, high-density lipoprotein cholesterol; LDL-C, low-density lipoprotein cholesterol; FBG, fasting blood glucose; CVD, cardiovascular disease. Triglycerides*: Median (Interquartile Range).

### Analysis of new carotid plaque formation for different baseline ba-PWV levels

A total of 738 Chinese participants were included in our analysis. After a mean 2.3-year follow-up, 230 of them developed carotid plaques. The prevalence of new carotid plaque formation was 36.5% in the PWV ≥1,400 cm/s group, which was significantly different from the prevalence of 21.2% in the PWV < 1,400 cm/s group. Table 2 shows the association between ba-PWV and the observation of new plaques on carotid ultrasound. These associations were significant with ORs (95% CIs) of 1.01 (1.00–1.02) for every 10-cm/s in the ba-PWV after adjusting for confounding factors. Furthermore, a ba-PWV greater than or equal to 1,400 cm/s has predictive value for the risk of new carotid plaque formation, with a 114% increased risk (95% CI: 1.50 to 3.03, P < 0.01) compared to that of the group with a ba-PWV less than 1,400 cm/s. This result remained (OR = 1.52, 95% CI: 1.02 to 2.25, P = 0.04) after adjusting for multiple traditional risk factors. We further put LDL-C, HDL-C, TG and FBG into analysis as confounding factors instead of dyslipidemia and diabetes mellitus, and the results did not change significantly (Supplementary Table 1).

**Table 2:** Different risk classifications in the prediction of new carotid plaque formation

Variable		Crude model		Adjusted model*	
		OR (95% CI)	P value	OR (95% CI)	P value
PWV (every 10 cm/s increase)	230 (31.3)	1.02 (1.01–1.02)	<0.01	1.01 (1.00–1.02)	0.03
PWV < 1,400	55 (21.2)	reference		reference	
PWV ≥ 1,400	175 (36.5)	2.14 (1.50–3.03)	<0.01	1.52 (1.02–2.25)	0.04

^*^Adjusted for age, sex, hypertension, dyslipidemia, diabetes mellitus, tobacco intake, alcohol intake, BMI, SCR, anti-hypertensive medicine, lipid-lowering medicine, anti-diabetic medicine and self-reported CVD history.

### Tests for interactions between subgroups

**Figure 1:**
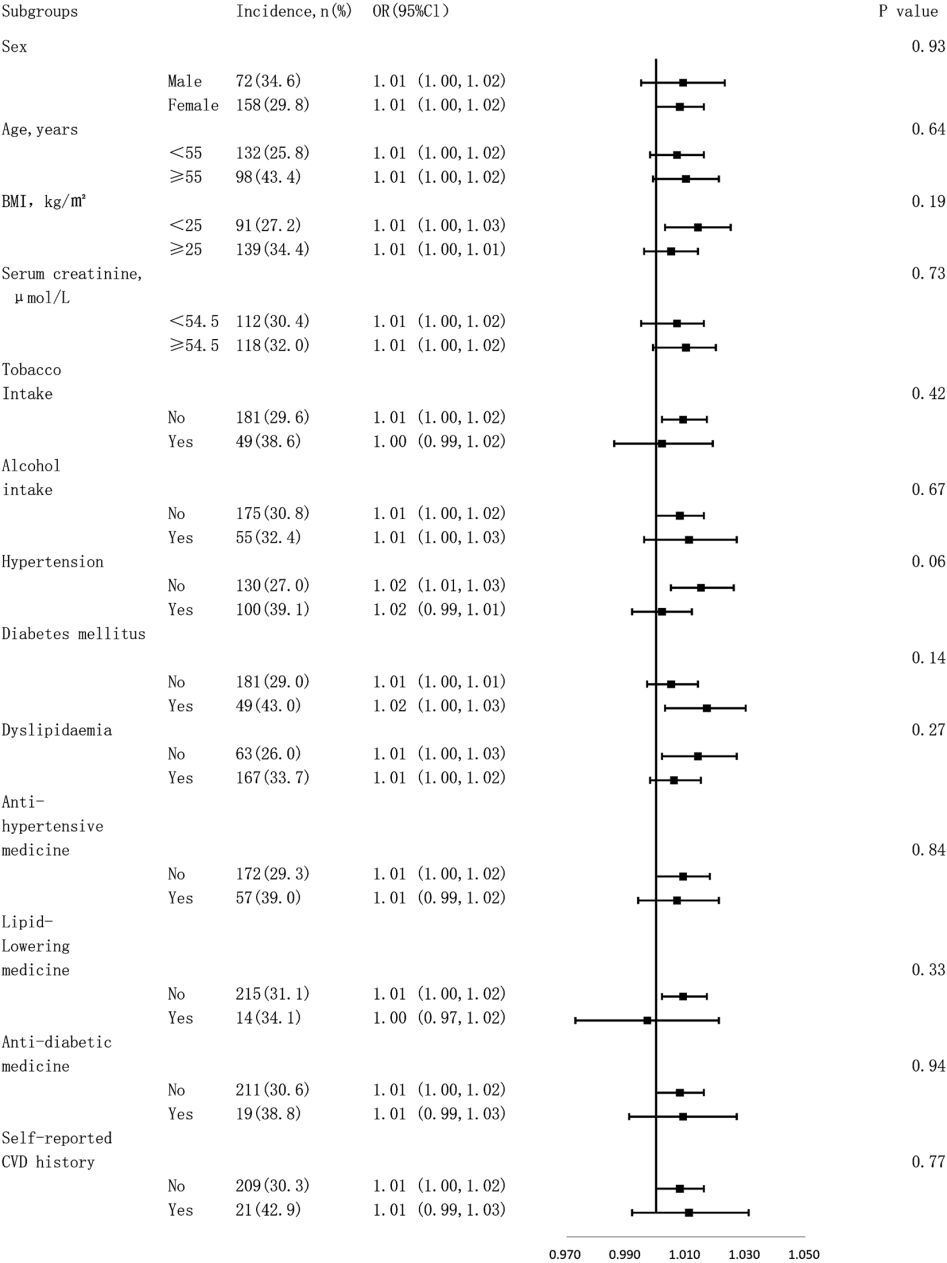
Subgroup analysis for the effect of ba-PWV on risk of carotid plaque formation after adjusting for confounding factors (Multivariate odd ratios [OR] and 95% confidence intervals [CI] are shown according to a ba-PWV value increase of 10 cm/s).

Figure 1 shows tests for interactions between the subgroups after adjusting for confounding factors. We found no significant heterogeneity among analysed subgroups according to sex (male or female), age (<55 or ≥55 years old), BMI (<25 or ≥25 kg/m^2^), SCR (<54.5 or ≥54.5 μmol/L), tobacco intake, alcohol intake, hypertension, diabetes mellitus, dyslipidemia, anti-hypertensive medicine, lipid-lowering medicine, anti-diabetic medicine or self-reported CVD history.

### Threshold effect analysis for the predicting effect of ba-PWV on carotid plaque formation

**Figure 2:**
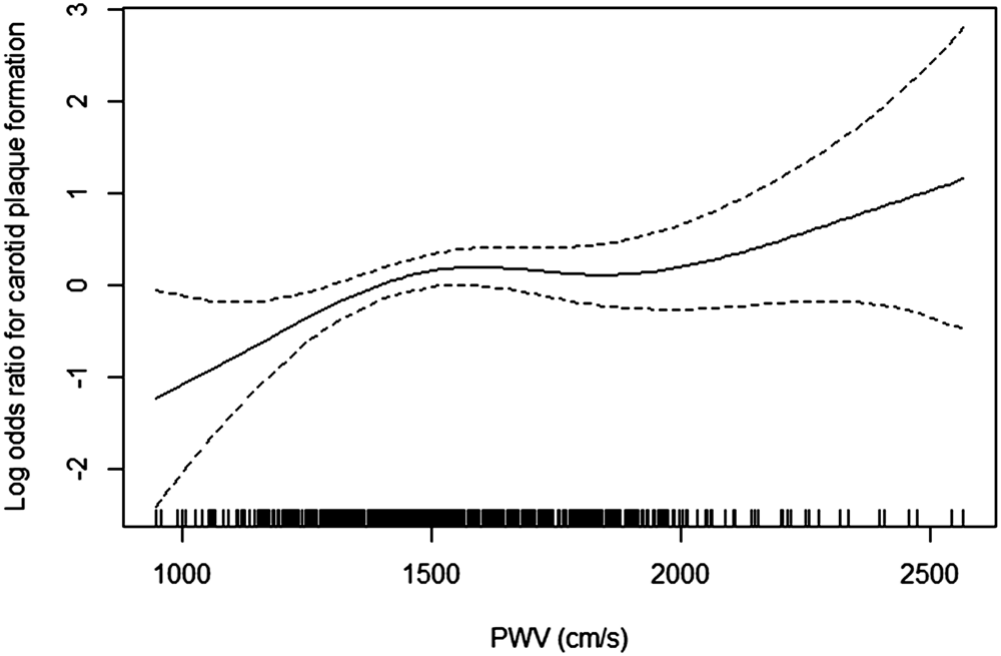
Smooth curve of the trend of the relationship between carotid plaque formation and the ba-PWV.

Figure 2 shows the trend of the relationship between carotid plaque development and the ba-PWV. Generally, a higher PWV corresponded to a higher OR for carotid plaque formation. However, the trend of the OR for carotid plaques was non-linear. The cut-off value was approximately 1,400 cm/s, which was consistent with the report by Yamashina *et al*.^17^ (Fig. 2).

We further conducted a threshold effect analysis using the cut-off value of 1,400 cm/s and found that in subjects with a ba-PWV < 1,400 cm/s, the risk of new carotid plaque formation increased with increasing baseline ba-PWV (OR = 1.04, 95% CI: 1.01 to 1.06, P = 0.01). In patients with a ba-PWV ≥1,400 cm/s, the risk of new carotid plaque formation was not significantly associated with ba-PWV (OR = 1.00, 95% CI: 0.99 to 1.01, P = 0.51). A log likelihood ratio test indicated significant differences between the two slopes below and above 1,400 cm/s (p = 0.03). (Table 3). Further analysis was done by adjusting LDL-C, HDL-C, TG and FBG instead of dyslipidemia and diabetes mellitus, and the results did not change significantly (Supplementary Table 2).

**Table 3:** Threshold effect analysis of baseline ba-PWV on the risk of new carotid plaque formation using piecewise linear regression model.

Model	Result [OR* (95%CI)]	P value
Model I one-line	1.01 (1.00, 1.02)	0.03
Model II turning point: 1400 cm/s		
Slope 1: ba-PWV<1400 cm/s	1.04 (1.01, 1.06)	0.01
Slope 2: ba-PWV≥1400 cm/s	1.00 (0.99, 1.01)	0.51
Slope 2 – Slope 1	0.97 (0.94, 1.00)	0.04
A log likelihood ratio test		0.03

^*^OR, odds ratio, represented the effect for every 10 cm/s increase of ba-PWV. Adjusted for age, sex, hypertension, dyslipidemia, diabetes mellitus, tobacco intake, alcohol intake, BMI, SCR, anti-hypertensive medicine, lipid-lowering medicine, anti-diabetic medicine and self-reported CVD history.

## Discussion

There were two main findings in this study. In a Chinese community-based population, a ba-PWV greater than or equal to 1,400 cm/s has predictive value for the risk of new carotid plaque formation, with a 52% increased risk (P = 0.04) compared to that of the group with a ba-PWV less than 1,400 cm/s, after adjusting for multiple traditional risk factors. Among subjects with a baseline ba-PWV less than 1,400 cm/s, the risk of new carotid atherosclerotic plaque formation increases in subjects with higher baseline ba-PWV. In contrast, such risk is not significantly associated to baseline ba-PWV in subjects with a ba-PWV greater than or equal to 1,400 cm/s.

The carotid artery and its branches are predilection sites of atherosclerotic plaque. As the upstream vessel of the cerebral artery, carotid plaque rupture can easily trigger cerebral ischaemic stroke, and the stiffness of the carotid artery is directly related to the cerebral blood flow. Observation of a new carotid artery plaque by carotid ultrasonography marks the occurrence and exacerbation of carotid atherosclerotic disease. Studies have shown that the predictive value of carotid plaques for cardiovascular events exceeds the predictive value of cIMT^18^.

The development of vascular disease is a long and complex process. Previous studies have discussed the relationship between arterial stiffness and atherosclerosis. Palombo *et al*. recently published a research article on this topic and reported that there were similarities between the two conditions in terms of risk factors and the site of occurrence^19^. Arterial stiffness could be related to arterial atherosclerosis through several mechanisms and pathways involving mechanical force on the inner wall of the blood vessels, an extracellular matrix disorder, endothelial dysfunction, elevated endothelial permeability and vascular ageing; however, a clear conclusion has not been reached^19^.

The ba-PWV and Carotid-femoral PWV (cf-PWV) are well accepted methods in clinical practice^20^. The ba-PWV is an index combining elastic and muscular peripheral arterial stiffness and is useful for cardiovascular outcome predictions in Asian populations^21^. By contrast, cf-PWV represents the stiffness of elastic arteries and commonly used in many European and American studies, but its measurement requires a certain level of operator experience. Conversely, ba-PWV is measured using standardized cuffs, which decreases the influence of operator technique on the measurement results.

An elevated ba-PWV indicates poorer arterial elasticity and higher arterial stiffness. The literature describing the relationship between the ba-PWV and carotid plaques is limited, especially for the Chinese population. Kobayashi *et al*. reported that the ba-PWV was associated with the presence of carotid atherosclerotic plaques in a cross-sectional study^22^. In an article by Joo *et al*., the ba-PWV was significantly correlated with compositecarotid and coronary atherosclerosis^23^ in a middle-aged asymptomatic population. Several studies have focused on the relationship between the ba-PWV and carotid atherosclerosis within specific populations. Munakata *et al*. found that the ba-PWV was a risk factor for carotid plaques among patients with end-stage renal disease^24^. Similar results were found by Masugata *et al*.^25^ among patients with type 2 diabetes and by Kubozono *et al*.^18^ among the male population.

Yamashina *et al*. conducted a study in 10,828 subjects and recommended a ba-PWV of 1,400 cm/s as a diagnostic cut-off value for atherosclerosis^17^. Accordingly, we used 1,400 cm/s as the demarcation point for the baseline ba-PWV, and for the first time confirmed the relationship between the baseline ba-PWV and new carotid plaque formation in a community-based cohort. The multivariate analysis showed that an elevated ba-PWV equal to or higher than 1,400 cm/s was an independent risk factor for the formation of new carotid atherosclerotic plaques. In the group with a baseline ba-PWV less than 1,400 cm/s, 21.2% of the subjects suffered from carotid plaques, whereas 36.5% of the subjects in the group with a baseline ba-PWV equal to or higher than 1,400 cm/s suffered from carotid plaques. This result may imply that arterial stiffness represented by ba-PWV precedes the occurrence of carotid atherosclerotic disease.

The results of this study also showed that the risk of new carotid plaque formation was elevated along with the baseline ba-PWV in subjects whose ba-PWV was less than 1,400 cm/s. This finding may indicate a lower ba-PWV value for the prediction of atherosclerotic diseases. The results also indicate that the risk of atherosclerotic disease may be increased even in populations with a ba-PWV previously considered normal, which emphasized the importance of primary prevention. Reducing ba-PWV levels may be effective for the prevention of carotid plaque formation and then the reduction of atherosclerotic disease sequentially.

A number of traditional risk factors have been shown to be associated with atherosclerotic disease, of which glucose and lipid metabolism disorders have particularly significant impact on atherosclerosis process. Dyslipidemia is usually considered to reflect the abnormalities of multiple laboratory indicators including LDL-C, HDL-C, TC and TG, and diabetes can represent the abnormalities of FBG and postprandial blood glucose. With this in mind, we used both continuous variables of LDL-C, HDL-C, TG, and FBG instead of dyslipidemia and diabetes mellitus in our data analysis, and the results showed no significant difference.

The current study has several limitations. First, previous studies have shown that ba-PWV is affected by blood pressure, and it is higher in hypertensive patitents^26^. However, the findings remained significant after adjusted hypertension status and antihypertensive treatment as the possible confounding factors. Furthermore, we’ve conducted a stratified analysis and found no interactions. Second, we have not yet observed enough endpoint events due to relatively short follow-up and a relatively young population, so we used carotid plaque, a well-accepted surrogate endpoint of cardiovascular disease, to investigate the relationship between arterial stiffness and atherosclerosis. A more solid conclusion need an increased sample size and an extended follow-up time. Finally, our conclusion, drawn from a Chinese community-based population, may not necessarily fit other population.

## Conclusion

The results of this study suggested that the baseline ba-PWV was independently associated with the risk of carotid plaques in a Chinese community-based population. The ba-PWV should be screened for the early prevention of atherosclerosis, even in subjects without arterial stiffness.

## Materials and Methods

### Study population

Subjects from an atherosclerosis cohort in the Gucheng and Pingguoyuan communities of Shijingshan District in Beijing, China were enrolled in this study^27^. In brief, a total of 3,823 participants aged ≥40 years at baseline survey from December 2011 to April 2012 were followed up on-site from May 2014 to July 2014. Among them, 1077 participants had available data for both baseline and follow-up quantitative carotid artery measurements, and baseline ba-PWV. Subjects with carotid plaques at baseline were excluded, and thus, a total of 738 subjects were ultimately included in our analysis. This study was approved by the ethics committee of Peking University First Hospital. The procedures followed were in accordance with institutional guidelines and with the principles of the Declaration of Helsinki, and each participant provided written informed consent.

### Data collection

Using a standard operating procedure, trained research staff conducted the anthropometric measurements and collected the baseline data. We used a standardized questionnaire to interview all participants and collect information, including the sociodemographic status, education, occupation, diet, lifestyle, health behaviour and medical history.

A venous blood sample was obtained from the forearm of each participant after an overnight fast of at least 12 hours. These samples were used to measure the TC, HDL-C, LDL-C, TG and FBG levels as well as the SCR level. A Cobas 8000 modular analyser series (Roche Diagnostics, Indianapolis, IN, USA) was used for all laboratory variable measurements at baseline in the laboratory of the Peoples Liberation Army General Hospital.

Hypertension was defined as either self-reported hypertension history, a SBP ≥140 mmHg or DBP ≥ 90 mmHg, or taking anti-hypertensive medicine. Diabetes mellitus was defined as either self-reported diabetes history, an FBG level ≥7.0 mmol/L, an OGTT value ≥11.1 mmol/L, or taking anti-diabetic medicine. Dyslipidemia was defined as any self-reported history of hyperlipidemia, a TG level ≥1.70 mmol/L, a TC level ≥5.18 mmol/L, an LDL-C level >3.37 mmol/L, an HDL-C level <1.04 mmol/L, or taking lipid-lowering medicine. CVD was defined as any self-reported suffering from coronary heart disease, stroke, or previous transient ischaemic attack. Current drinking was defined as drinking at least once per week for at least half a year and current smoking was defined as smoking at least one cigarette per day for at least half a year. We used weight (kg) divided by height (m) squared to calculate the body mass index (BMI). The peripheral blood pressure was measured using the standard method, and the average of 3 measurements was used in the analysis^27^.

### Carotid ultrasonography

A trained and certified sonographer performed carotid ultrasonography on all participants using the GE Vivid i ultrasound system (GE Medical Systems, Milwaukee, WI, USA). The system was equipped with an 8-MHz linear array vascular probe. Carotid ultrasounds were performed according to the standard scanning and reading protocols^28^. A carotid plaque was defined as a focal structure encroaching into the arterial lumen by at least 50% more than into the surrounding tissue with a carotid intima-media thickness (cIMT) value of 0.5 mm or >1.5-mm thickness from the intima-lumen interface to the media–adventitia interface at the level of the internal carotid artery, common carotid artery and bifurcation. The presence of a plaque was judged by certified sonographers.

### Brachial-ankle Pulse Wave Velocity

We used an Oscillometry-based device (BP-203RPE III; Colin-Omron, Co., Ltd., Tokyo, Japan) to measure the ba-PWV. Each subject was laid in the supine position with PWV cuffs wrapped around both ankles and upper arms. Trained research staff recorded the pulse waveforms after the subject rested for at least five minutes, and the highest ba-PWV was recorded for analysis after examination of both the left and right sides.

### Statistical analysis

We expressed the descriptive statistics as frequencies and percentages for nominal variables, mean ± standard deviation (s.d.) for normally distributed continuous variables and median (interquartile range) for skewed distributed ones. We used Student’s t-test to compare the successive variables with a normal distribution whereas Kruskal Wallis test for skewed distributed ones, and Pearson’s χ^2^-test was applied to compare the incidence of all categorical variables between groups. Multivariate logistic regression models were used to investigate the association of the ba-PWV with new carotid plaque formation after adjusting for age, sex, hypertension, dyslipidemia, diabetes mellitus, tobacco intake, alcohol intake, BMI, SCR level, anti-hypertensive medicine, lipid-lowering medicine, anti-diabetic medicine and the self-reported CVD history. In this study, a P value <0.05 (two-sided) was considered as significant different for all tests. All statistical analyses were performed using Empower(R) (www.empowerstats.com, X&Y solutions, Inc., Boston, MA, USA) and the R software (http://www.R-project.org).

### Data availability

The datasets generated during and/or analysed during the current study are not publicly available due to protection of subject privacy but are available from the corresponding author upon reasonable request.

## Electronic supplementary material

Supplementary Tables
