# Supplementary material for: Brachial-Ankle Pulse Wave Velocity is Associated with the Risk of New Carotid Plaque Formation: Data from a Chinese Community-based Cohort
Source: Sci Rep. 2018 May 4;8:7037. doi: 10.1038/s41598-018-25579-2 (PMC5935681; doi:10.1038/s41598-018-25579-2)
Supplement: Supplementary file 1 — Supplementary Tables [file 41598_2018_25579_MOESM1_ESM.pdf]

# Brachial-Ankle Pulse Wave Velocity is Associated with the Risk of New Carotid Plaque Formation: Data from a Chinese Community-based Cohort

Yao Yang[1], PhD, Fangfang Fan[1], MD, Minghao Kou[2], Ying Yang[1], MD, Guanliang Cheng[1], PhD, Jia Jia[1], MPH, Lan Gao[1], MD, Zechen Zhou[2], Dafang Chen[2], PhD, Yan Zhang[1*], MD, Yong Huo[1*], MD

Corresponding author: Yan Zhang[1], MD, Yong Huo[1], MD

1. Department of Cardiology, Peking University First Hospital, Beijing, China
2. School of Public Health, Peking University Health Science Centre, Beijing China

**Correspondence and reprint requests should be addressed to:**

Yan Zhang, MD

Department of Cardiology, Peking University First Hospital, Beijing, China

Address: No. 8 Xishiku Street, Xicheng District, Beijing, China

Email: drzhy1108@163.com

Phone: 86-10-83572283

Fax: 86-10-66137748

Yong Huo, MD

Department of Cardiology, Peking University First Hospital, Beijing, China

Address: No. 8 Xishiku Street, Xicheng District, Beijing, China

Email: huoyong@263.net.cn

Phone: 86-10-83572283

Fax: 86-10-66137748

Supplementary Table 1 Different risk classifications in the prediction of new carotid plaque formation

| Variable | | Crude model | | Adjusted model* | |
|---|---|---|---|---|---|
| | | OR (95% CI) | P value | OR (95% CI) | P value |
| PWV (every 10 cm/s increase) | 230 (31.3) | 1.02 (1.01-1.02) | <0.01 | 1.01 (1.00-1.02) | 0.02 |
| PWV <1,400 | 55 (21.2) | reference | | reference | |
| PWV ≥1,400 | 175 (36.5) | 2.14 (1.50-3.03) | <0.01 | 1.56 (1.04-2.33) | 0.03 |

* Adjusted for age, sex, hypertension, high-density lipoprotein cholesterol, low-density lipoprotein cholesterol, triglycerides, fasting blood glucose, tobacco intake, alcohol intake, BMI, SCR, anti-hypertensive medicine, lipid-lowering medicine, anti-diabetic medicine and self-reported CVD history.

Supplementary Table 2 Threshold effect analysis of baseline ba-PWV on the risk of new carotid plaque formation using piecewise linear regression model

| Model | Result [OR* (95%CI)] | P value |
|---|---|---|
| Model I one-line | 1.01 (1.00, 1.02) | 0.02 |
| Model II turning point: 1400cm/s | | |
| Slope 1: ba-PWV<1400cm/s | 1.04 (1.01, 1.07) | <0.01 |
| Slope 2: ba-PWV≥1400cm/s | 1.00 (0.99, 1.01) | 0.47 |
| Slope 2 – Slope 1 | 0.97 (0.94, 1.00) | 0.03 |
| A log likelihood ratio test | | 0.03 |

* OR, odds ratio, represented the effect for every 10 cm/s increase of ba-PWV. Adjusted for age, sex, hypertension, high-density lipoprotein cholesterol, low-density lipoprotein cholesterol, triglycerides, fasting blood glucose, tobacco intake, alcohol intake, BMI, SCR, anti-hypertensive medicine, lipid-lowering medicine, anti-diabetic medicine and self-reported CVD history.
